# The Action and Uses of Digitalis

**Published:** 1880-01-20

**Authors:** A. G. Hobbs

**Affiliations:** Indiana


### Southern Medical Record.

EDITORS:

T. S. POWELL, M.D.  W. T. GOLDSMITH, M.D.  R. C. WORD, M.D.

*R. C. WORD, M.D., Managing Editor.*

☞All Communications and Letters on Business connected with the RECORD must be addressed to the Managing Editor.

VOL. X.  ATLANTA, GA., JANUARY 20, 1880.  NO. 1.

### ORIGINAL AND SELECTED ARTICLES.

### *THE ACTION AND USES OF DIGITALIS.*

BY A. G. HOBBS, M.D., OF INDIANA.

When digitalis is gradually introduced into the system, three stages are observed.

1. The frequency of the pulse diminishes, and the arterial pressure rises.

2. The frequency of the pulse is still more diminished, but the arterial pressure falls.

3. The frequency of the pulse rises, and the arterial pressure falls still lower.

The action, though, that we more frequently observe, especially from large doses, is that in which the pulse is lessened in frequency but rendered fuller. This is the action that we generally seek, especially in cardiac and inflammatory troubles.

According to Binz, digitalis has a special stimulant action upon the pneumogastric nerve in its whole extent, from its origin to its terminal fibres in the heart, directly exciting the muscles of the heart to action. This explains the *modus operandi* of its action in opium poisoning, where death is imminent from the stoppage of the heart's action.

Lethal doses have a directly opposite action upon the heart, that of paralyzing its action. If it be given in inflammatory diseases accompanied by fever, not only does the pulse become less frequent, but the temperature becomes lower, the local inflammation is thus cut short, said by some to be aborted.

Physiological experimentations have proven that digitalis lessens the calibre of the arterioles. When the smaller arteries are lessened in calibre, the larger ones necessarily become fuller; thus is the empirical fact physiologically explained, that digitalis renders the pulse fuller. A lethal dose is followed by a transitory rise of temperature, which is soon succeeded by a marked and sustained reduction. This action is explained by Ackerman thus :

Owing to the increased resistance from the diminution of the calibre of the arterioles, the actual energy expended by the heart is first converted into heat till the heart is toned down, as it were, and becomes accustomed to the resistance. Then the slowing of the circulation hinders the combustion process in the lungs, and the increased arterial pressure facilitates the dissemination of heat from the surface, consequently the temperature falls.

On the other hand, when extremely small doses are given, the first sensation is that of coldness, followed in a few hours by a rise in temperature; this is supposed to be due to vaso-motor irritation.

It is thought, from its physiological action, that digitalis should have a somewhat similar action to ergot on the enlarged uterus, that of stimulating to energetic contraction its muscular fibre, and thus acting as a valuable remedy in uterine hemorrhage. By its quality of causing the contraction of the arterioles, it diminishes the blood supply to the erectile tissue of the penis, and thus acts as an anaphrodisiac.

It has always been thought that the diuretic action of digitalis was indirect, due to its action upon the heart, increasing the blood pressure and thereby stimulating the process of osmosis in the kidneys; but Lander Bronton has recently demonstrated that its diuretic action is due to a special action upon the malpighian tufts.

This agent perhaps has a cumulative effect which is due to the retention of digitaline in the circulation. This is an effect never observed, however, except when the dose is so immoderately large that the kidneys cannot eliminate it all.

But this is no unique action of digitalis, as it was thought twenty years ago; any other powerful remedy, given in sufficiently large doses and continued a sufficient length of time, will display the same phenomenon.

Digitalis lengthens the period of systol, tones the heart and strengthens it. This is done partly by a direct action upon the heart's muscle, and partly by stimulating the cardiac inhibitory fibres of the vagus, which, when stimulated, hold more forcibly in check the rapid beat produced by the sympathetic.

Digitalis was formerly called a heart sedative instead of a heart tonic, because it controlled or sedated those violent palpitations or flutterings of the heart which were supposed to be due to overaction, when in reality they were typical symptoms of a weakened heart—a heart that had a blood current to propel, and had not the propulsive force, like a locomotive starting up a grade without sufficient steam to overcome the momentum of the train; it exhausts its power by frequent and ineffectual strokes, the engineer puts on more steam, that is gives it more power; its strokes become slower and more regular, and the train moves on.

*Uses.*—In those violent and ineffectual contractions of the heart which follow carditis, its action is marked.

In that ordinary affection known as palpitation, which is so often due to sympathy with some other organ, or to some neurosis affecting the guiding nerves of the heart's action, especially the vagus, digitalis is very useful.

If it be true, as we have every evidence to believe from physiological investigation, that it diminishes the calibre of the smaller arteries, it is indicated in hemorrhages particularly those due to degenerative changes in the lungs and in capillary hemorrhage of any kind, especially where the hemorrhagic diathesis is displayed. Upon this hypothesis I always combine it in my prescriptions for chronic gleet, spermatorrhea, etc., where an erect penis is detrimental to an early cure.

Digitalis is not suitable where a rapid effect is desired. For instance, Traube claims that its antipyretic action does not show itself until from 30 to 60 hours after it has been first used.

It is useful in hæmoptisis accompanied by fever, especially where bloody mucus is spit.

It has been used in post partum hemorrhage, but its action is so slow that I should hesitate to confide much in it.

Some claim that it has acted like a charm in menorrhagia.

In using it in all the above cases where the *rationale* of its indication is founded upon its property of contracting the arterioles, I should always combine it with ergot.

Its utility in scarlet fever is without doubt; it lowers the temperature and maintains the action of the kidneys, thus obviating the two principal sources of danger in that disease.

It is recommended highly in rheumatic fever, but my scanty observation does not confirm its beneficial effects.

There is considerable evidence that it is serviceable in pneumonia; in fact, it has its several advocates as a useful remedy in each and all of the inflammatory affections.

Its greatest field of usefulness is in the cardiac troubles. Bartholow says that in general terms it is indicated when the action of the heart is rapid and weak, and the arterial tension low, and is contraindicted when the action of the heart is vigorous, and the arterial tension high. In simple hypertrophy and stenosis, where both are compensatory, digitalis is worse than useless.

It is valuable in all heart lesions, where there is want of compensation, by its action upon that organ in restoring the mechanical balance of the circulation.

It may be put down as a general rule never to administer digitalis where simple hypertrophy of the ventricular walls exists. When the hypertrophy is being toned down by fatty degeneration, and secondary dilatation is taking place, digitalis may be called for as a temporary relief, but in this case it, nor anything else, can afford the poor sufferer any permanent relief.

Many practitioners, without studying closely the action of digitalis, conclude that it is indicated wherever and whenever they find cardiac diseases ; in fact, they will administer it in all heart troubles, whether organic or functional, in nervous palpitations or in valvular lesions.

Suppose we take two hypothetical cases : First, we have a laboring but weak and frequent heart beat; the pulse is soft and the artery is incompletely filled at each pulsation; the patient becomes weak and loses muscular tonicity; as a consequence, the arterioles dilate, and the blood passes on to the veins' side of the circulation, but, after reaching the right ventricle and thence the lungs, it cannot rapidly re-enter the left ventricle because of its dilatation and consequent loss of propelling power ; thus is there a congestion of the lungs brought about. But suppose we administer digitalis in this case. The heart is toned and strengthened, an hypertrophy of the ventricular walls is induced to compensate the dilatation, and secondarily the arterioles are contracted. The blood is now propelled through the aorta with more force, and the pulmonary veins can empty their blood into the left ventricle more rapidly and thereby relieve the pulmonary congestion and restore an equilibrium to the circulation.

In the second hypothetical case we have a full, strong heart-beat; the pulse is firm and cord-like; the pulsation of every superficial artery is plainly visible. These symptoms are intensified by the febrile condition, and, if allowed to continue, will perhaps induce arteritis, or it may be congestion of the brain, from the imminent volume of blood thrown into the weak-coated cerebral arteries. Hypertrophy already exists in this case, and digitalis is certainly contra-indicated because we do not wish the already dangerous condition intensified. Aconite will act well in this case, if not continued too long. In fact, where digitalis is indicated in cardiac diseases, aconite is contra-indicated, and *vice versa*.

Many experimenters have advocated the use of digitalis in nervous diseases, such as acute maniacal delirium, chronic mania, delirium tremens, etc., etc. Combined with iod. pot. it was the great Trousseau's treatment for exophthalmia goitre.

It is a generally useful remedy in dropsy, and an especial one in dropsy of valvular lesions and renal dropsy from acute desquamative neprhitis.

In opium poisoning it should always be given in connection with atropia to prevent failure of the heart's action.

This very extensively applicable remedy has its advocates as a special remedy in other diseases that I have not mentioned. In fact, it is one of the most universally useful remedies in our armamentarium.

